# Interactions between Phenolic Acids, Proteins, and Carbohydrates—Influence on Dough and Bread Properties

**DOI:** 10.3390/foods10112798

**Published:** 2021-11-13

**Authors:** Simone Schefer, Marie Oest, Sascha Rohn

**Affiliations:** 1 Institute of Food Chemistry, Hamburg School of Food Science, University of Hamburg, Grindelallee 117, 20146 Hamburg, Germany; simone.schefer@chemie.uni-hamburg.de (S.S.); marie.oest@chemie.uni-hamburg.de (M.O.); 2 Department of Food Chemistry and Analysis, Institute of Food Technology and Food Chemistry, Technische Universität Berlin, TIB 4/3-1, Gustav-Meyer-Allee 25, 13355 Berlin, Germany

**Keywords:** phenolic acids, proteins, carbohydrates, interactions, bread and dough properties

## Abstract

The understanding of interactions between proteins, carbohydrates, and phenolic compounds is becoming increasingly important in food science, as these interactions might significantly affect the functionality of foods. So far, research has focused predominantly on protein–phenolic or carbohydrate–phenolic interactions, separately, but these components might also form other combinations. In plant-based foods, all three components are highly abundant; phenolic acids are the most important phenolic compound subclass. However, their interactions and influences are not yet fully understood. Especially in cereal products, such as bread, being a nutritional basic in human nutrition, interactions of the mentioned compounds are possible and their characterization seems to be a worthwhile target, as the functionality of each of the components might be affected. This review presents the basics of such interactions, with special emphasis on ferulic acid, as the most abundant phenolic acid in nature, and tries to illustrate the possibility of ternary interactions with regard to dough and bread properties. One of the phenomena assigned to such interactions is so-called dry-baking, which is very often observed in rye bread.

## 1. Introduction

In complex plant-based foods, a large number of different compounds come into contact with each other; either already in the plant matrix or during food-processing. These compounds can interact depending on their chemical-structural characteristics, the processing methods used, and on reaction conditions. Interactions influence the functional properties of all interaction partners. Consequently, food quality and safety are affected.

In this context, phenolic compounds (PCs) are of special interest due to their high abundance in complex plant-based foods and their susceptibility for reactions depending on their diverse chemical structures. Thus, various kinds of interactions are possible, with potential impacts on organoleptic attributes, as well as the nutritional-physiological properties of foods [1,2].

The main function of PCs in plants is believed to be protection from exogenous biological or physical stress factors, such as pests or UV-light, but they are also the basis for the stability of fibers, especially in grasses. However, further important functions come into focus in plant-based food products, such as chemical and physical (e.g., antioxidant capacity and color) properties, as well as nutritional-physiological impacts (e.g., bitter or astringent taste, but also a number of different health-benefits) [3,4]. PCs’ antioxidant activity, being a distinct chemical property, makes them valuable food ingredients, too. They inhibit various decomposition reactions, but also form the basis for promoting various health benefits, most of which are believed to be based on the neutralization of free radicals present in the human organism. PCs are further ascribed with anti-inflammatory properties through the regulation of cellular processes, as well as providing antimicrobial activity. Some studies even link PCs to anti-carcinogenicity in connection to the above-mentioned properties [5,6,7,8].

Regarding the interactions of PCs, it has to be considered that these consist of a broad variety of different subgroups. Among these, phenolic acids (PAs) are quite interesting, as they are found in fairly high amounts in many food plants. Their ratio to the other food compounds is in a range, where they might significantly interact with the major food constituents. Therefore, interactions with proteins or polysaccharides are worth studying, as these interactions determine factors like the texture of a food, or are responsible for the biological value (e.g., indispensable amino acids) [9]. An interaction between those compounds might severely affect food quality.

PCs can interact covalently or non-covalently with biopolymers. In contrast to the mostly reversible non-covalent interactions, covalent interactions are almost irreversible. Oxidation of PAs leads to the formation of quinones, which form the basis for Michael-type addition reactions, leading to covalently bound adducts. Although highly dependent on chemical-structural characteristics, the chemical environment is also a major determinant for the kind of interactions that can take place [10]. Processing conditions, such as temperature and time, availability of oxygen, etc., need to be considered as well [11,12,13,14,15,16].

The pH value plays a decisive role with regard to which type of interaction is favored. While non-covalent interactions mostly occur in an acidic to neutral environment, covalent interactions are favored during alkaline conditions. However, oxidation and. consequently, covalent interactions can also be induced enzymatically; either via peroxidases (POD) or polyphenoloxidases (PPO). It needs to be kept in mind that (oxidized) phenolic compounds preferably react with themselves to form complex brown polymers (melanins) [17].

In addition to the reactions of PAs with each other to form oligomers, proteins are particularly attractive interaction partners due to their nucleophilic character and their hydrophilic or hydrophobic regions. Interactions between proteins and PAs and their effect on the nutritional value of food and food function have been studied intensely in recent years [18,19,20,21,22,23,24]. The interactions of some proteins with PAs have an impact on physicochemical (e.g., protein solubility, isoelectric point, hydrophobicity), technofunctional (e.g., emulsifying, foaming, gelling), as well as nutritional properties (bioavailability, digestibility, allergenicity) of the proteins [25,26,27,28,29,30,31,32,33,34,35,36,37,38].

However, regarding their ability to interact in complex food matrices, PAs are not only known for their interactions with proteins, but also with another important biopolymer class: carbohydrates. These are particularly important, because of their diversity and high abundance in plant-based foods, but also because of their countless technofunctionalities in foods. Similar to the interactions with proteins, the occurrence of both covalent and non-covalent interactions with carbohydrates is possible. With regard to covalent interactions between polysaccharides and PAs, one of the most prominent examples is the crosslinking between arabinoxylans and ferulic acids in the plant order Poales. The crosslinking enables stabilization of the plant cell walls, tissues, and especially fibers. However, non-covalent interactions of PAs and polysaccharides were also observed to occur via complex formation in starch granules. All these interactions significantly affect physicochemical (e.g., solubility, gelling), technofunctional (e.g., starch swelling, degradation, and retrogradation), as well as nutritional properties (fiber digestibility, bioavailability of further nutrients) of the carbohydrates [3,39,40,41].

With regard to the above-mentioned aspects, both proteins and polysaccharides play a crucial role, especially in cereal-based products, such as bakery goods. These consist of more than 80% carbohydrates and proteins, and a content of PAs of up to 0.17%, depending on the cereal-type [42]. As PAs are able to interact with both compound classes, quality parameters of dough and bread (rheology, water binding capacity and availability, starch gelatinization, flavor, and taste) are significantly affected [3,39,40,41,43,44,45].

The aim of this review is to summarize the various possible interactions between PAs and proteins, as well as carbohydrates, with a special focus on cereal-based foods. In this context both naturally occurring interactions and interactions resulting from enrichment with PAs are considered. The following sections deal with the basic characteristics and chemical mechanisms, as well as the complex (ternary) interactions, and their effects on dough and bread.

## 2. Phenolic Acids

Phenolic acids (PAs) are found ubiquitously in almost all food plants, such as fruits, vegetables, cereals, etc., mostly ester-bound to carbohydrates in the form of *O*-glycosides [46,47,48]. Among the most important substances of the PAs are the hydroxybenzoic acids and hydroxycinnamic acids, which biosynthetically derive from cinnamic acid or *p*-coumaric acid by hydroxylation and methylation. Some of the most typical representatives of PAs include gallic acid, syringic acid, caffeic acid (CA), ferulic acid (FA), and sinapic acid [49]. In food, various reaction products of PAs, formed during enzymatic browning, can lead to undesirable changes in taste and color [49]. However, PAs act as antioxidants, scavenging free radicals, inhibiting lipid oxidation, and thus, represent an important additive in food and beverages [50].

**Figure 1:**
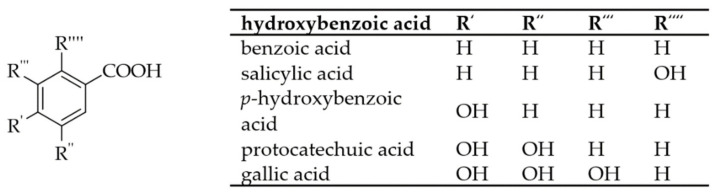
The structures of hydroxybenzoic acid derivates.

**Figure 2:**
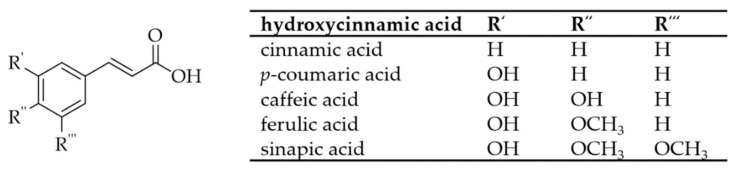
The structures of hydroxycinnamic acid derivates.

For the biosynthesis of PCs, the amino acid phenylalanine is the starting compound. It is synthesized in the shikimic acid pathway by plant organisms or microorganisms from 5-phosphoryl-D-erythrose and 2-phosphoenolpyruvate. Phenylalanine is converted to cinnamic acid by phenylalanine ammonia-lyases. Subsequently, monooxygenases can incorporate hydroxyl groups into the PCs to produce *p*-coumaric acid. Further enzymatic oxidation results in the formation of CA [51]. In plants, oxidative decarboxylation and β-oxidation of cinnamic acid produces hydroxybenzoic acids, which can also be derivatized by the monooxygenases into their derivatives [52]. Figure 1 and Figure 2 show a schematic representation of the different PAs.

**Figure 3:**
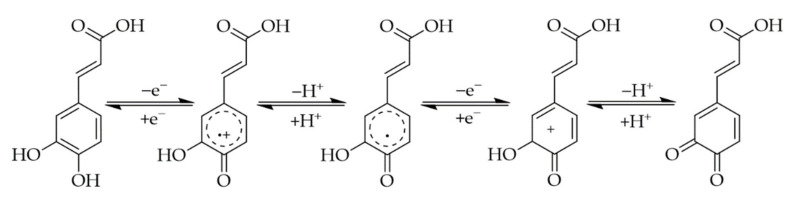
Structural representation of the *o*-quinone formation from CA.

**Figure 4:**
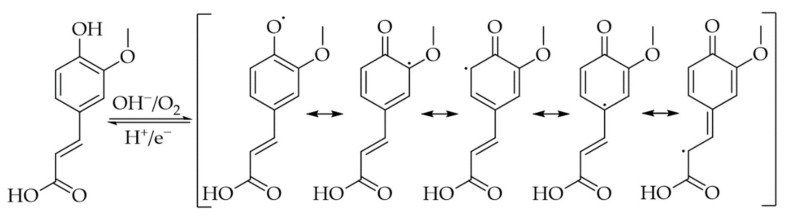
Formation of the *o*-semiquinone radicals of FA.

It is known that both the enzymatic and the non-enzymatic oxidation of PAs and the subsequent formation of quinones or semiquinones significantly increases the reactivity and binding affinity towards biomolecules [53]. In this context, the quinones are considered to be more reactive than the semiquinones. The oxidation of diphenols, such as CA, leads to the formation of a reactive and redox-active *o*-quinone (Figure 3), which is susceptible to reaction with nucleophiles due to its electrophilicity. However, in competition to this reaction, the *o*-quinones can also react with themselves, forming dark pigments—the melanins [20]. In the case of monophenolic derivatives, such as FA, only the single hydroxyl group and not the methoxy group can be oxidized, resulting in a semiquinone that cannot be further oxidized to a “full” quinone (Figure 4) [54,55]. Consequently, quinones and semiquinones form a variety of reaction products, which makes the elucidation of binding modes and mechanisms considerably more difficult [56].

**Figure 5:**
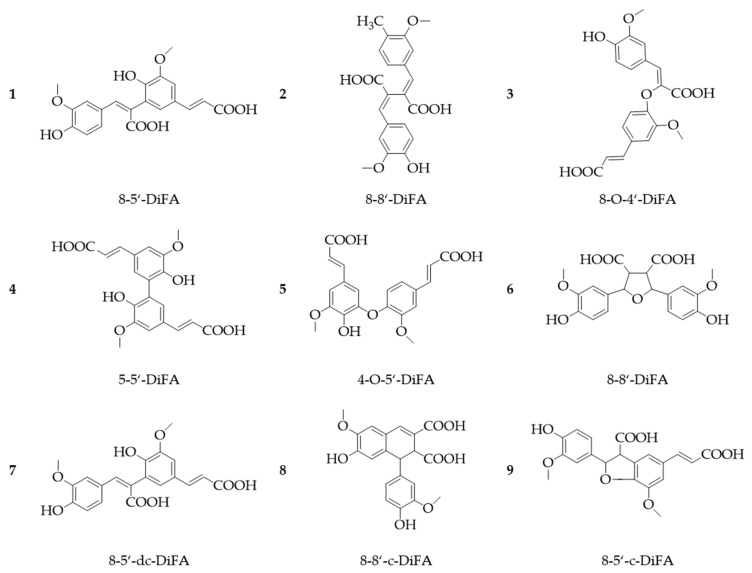
Chemical structures of selected ferulic acid dimers. (**1**): 8-5′-dehydrodiferulic acid (DiFA); (**2**): 8-8′-dehydrodiferulic acids; (**3**): 8-O-4′-dehydrodiferulic acid; (**4**): 5-5′-dehydrodiferulic acid; (**5**): 4-o-5′-dehydrodiferulicacid; (**6**): 8-8’-tetrahydrofuran (THF)-diferulic acid; (**7**): 8-8′-cyclic(c)-diferulic acid; (**8**): 8-5′-decarboxylated (dc)-diferulic acid; (**9**): 8-5′-cyclic-diferulic acid [58,59].

As PAs are easily oxidized and lead to radical formation, they are primarily highly reactive towards other radical components, resulting in a high antioxidant capacity [53]. In nature, PAs frequently occur as dimers, which are formed via a photochemical or a radical mechanism. This phenomenon has been observed particularly for FA [57]. For example, in grasses, FA and diferulic acids especially are frequently linked to the plant cell wall via ester bonds. The 8-5′-, 8-8′-, 8-O-4′- 5-5′-, and 4-O-5′-coupled diferulic acids are the most prominent coupling variants (Figure 5, Nos. 1–5). Further diferulic acids were identified (Figure 5, Nos. 6–10), which were released from the cell wall by hydrolysis after alkaline treatment [58,59,60]. In comparison to the monomeric FA, the dimers possess a higher radical scavenging activity and are therefore more reactive towards other compounds [61].

As a result of their reactive nature and their high abundance in plant-based foods, PAs are highly susceptible for further or subsequent interactions with other compounds, either already in the plant or during processing. In this context, proteins and carbohydrates are of particular interest as interactions partners, as they represent the main components of many food matrices and together determine the majority of the technofunctional and nutritional properties of plant-based foods.

## 3. Interactions of PAs with Proteins

Proteins are probably the most prominent reaction partners for PAs in food systems. The basis for the various properties of proteins is their structural diversity. Regarding interactions, they offer different potential interaction sites through hydrophobic and hydrophilic domains, as well as the nucleophilic side chains of selected amino acids.

As already mentioned, proteins have a high influence on sensory and functional, as well as nutritional, properties of foods. In this context, various attributes of proteins are important, and almost all depend on their three-dimensional structure. The primary sequence and the content of the single amino acids, as well as the number of disulfide bridges, play an important role regarding the protein’s conformation. During food processing, proteins either tend to form higher molecular weight aggregates, but also form networks, as, for example, the gluten-like network, or they are digested into peptides and amino acids. In both cases, the protein’s structure and the accessibility of certain amino acid side chains, representing reactive sites (for interactions), is crucial.

The interactions between proteins and PAs are affected by a number of different parameters: besides all factors influencing the protein structure directly (‘denaturation’), i.e., temperature, pH, salt, and protein concentration, the structure of the phenolic acid itself also has a tremendous impact on the kind of interactions that can take place [11,12,13,14,15,16]. The properties of the PAs affecting non-covalent interactions are: partition coefficients (log*p* values), keto-groups, double bonds (in the side chains), and the benzene ring [15].

Non-covalent interactions are mostly reversible and can take place via different types of mechanisms: hydrogen bonding, electrostatic interactions, hydrophobic interactions, van der Waals interactions, and π-bonding/π-π-stacking. These are quite labile and especially dependent on the pH value, ionic strength, and temperature. A low to neutral pH benefits the formation of non-covalent bonds, while ionic strength influences electrostatic interactions, depending on the protein’s isoelectric point. The temperature has a decisive influence on the formation of hydrogen bonds or hydrophobic interactions. While an increase in temperature leads to a decreased extent of hydrophilic interactions, there is an increase in the extent of hydrophobic interactions with increasing temperature. [11,12,13,16].

**Figure 6:**
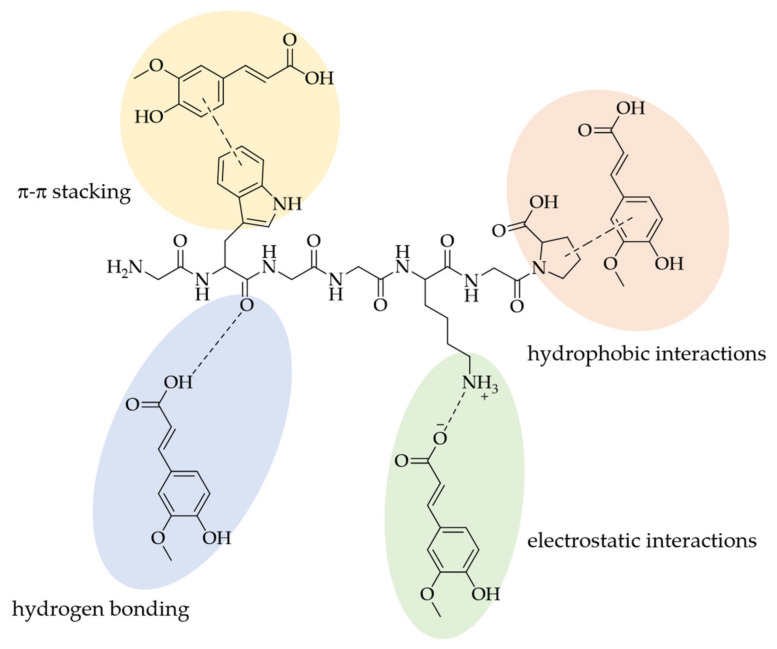
Possible non-covalent interactions between proteins and FA (as an example for almost all PAs).

Hydrophobic interactions of PCs with proteins take place at regions/domains of the protein where a high number of amino acid residues—of alanine, cysteine, glycine, methionine, proline, phenylalanine, leucine, isoleucine, and valine, all mostly adjacent to each other in the sequence—are present. Hydrogen bonds can occur with amino acid residues of asparagine, arginine, aspartic acid, cysteine, lysine, histidine, glutamine, glutamic acid, serine, threonine, tyrosine, and tryptophan [20,62,63]. Hydrogen bonds usually occur between the phenolic hydroxyl groups and the protein’s peptide bonds [64,65,66]. Figure 6 exemplarily shows the non-covalent interactions between proteins and PAs in the case of FA.

Regarding the influence of temperature on non-covalent interactions, Prigent et al. [13] found that the non-covalent interactions between chlorogenic acid (CGA) and bovine serum albumin (BSA) decreased with increasing temperature. At different concentrations of CGA, the number of bound molecules of CGA per molecule of BSA decreased by about 63–83% from 25 °C to 60 °C. The decreasing interaction with increasing temperatures suggests that hydrophilic interactions, such as hydrogen bonds, might be responsible for the interactions between CGA and BSA. Hydrophobic interactions likely play a minor role in the binding of CGA and BSA, as those would increase with increasing temperature [13]. These findings are in accordance with studies described by Ojha et al. [37], who suggested that the binding of FA to BSA at pH 7.4 is driven by hydrogen bonding and electrostatic interactions. The binding of FA also resulted in a change of the secondary structure of BSA. Additionally, increased binding of FA to BSA led to a higher thermal stability of the protein, as its melting temperature increased upon binding with FA.

In contrast, studies done by Rawel et al. [23] showed that the interactions of FA with different types of proteins also possess a hydrophobic character, being dependent on the pH value. They found that FA has a higher binding capacity (measured with the Hummel–Dreyer method) towards BSA, whey protein, and gelatin at a low pH of 4.8 in comparison to a neutral pH of 7.4. As a proline-rich protein, gelatin displays a high capacity for non-covalent hydrophobic interactions due to prolin’s interrupting the formation of secondary protein structures [24,67]. Furthermore, Rawel et al. [23] found that a decrease in pH (around the isoelectric point of BSA) also diminishes binding sites of BSA for FA and CGA, indicating the importance of hydrophobic interactions. This is supported by the fact that the affinity of CGA for BSA increases with increasing ionic strength [13]. However, in another study, Rawel et al. [68] found inverse results for the binding capacity of FA and BSA measured with the quenching of tryptophan fluorescence. That result is in line with the results described by Li et al. [69], Zhang et al. [70], and He et al. [71]. Only Ojha et al. [37] found a tenfold higher binding capacity. An overview of the different binding capacities is given in Table 1. Measurements made by Li et al. [69] showed, compared to FA, a higher binding capacity for CGA and CA, respectively, which might be due to the higher availability of hydroxyl groups capable to form hydrogen bonds with BSA. With rising temperature, they also found an increase in the binding capacity for FA as well as CGA and CA. This effect may occur because of temperature-dependent structural changes of BSA. At pH 7, BSA showed a loss of α-helical domains over a temperature of 30 °C and an increase of β- and disordered structure motifs [72]. This results in a higher availability of interaction sites for the PAs as a consequence of changes in protein conformation. Due to its structure, BSA has the ability to interact with both hydrophilic and hydrophobic compounds, but hydrophilic interactions seem to be stronger [73]. Table 1 shows an overview of the measured binding capacities (K-value) for FA and BSA and the methods used in each case.

Abdollahi et al. [74] reported non-covalent interactions between FA and β-lactoglobulin (β-LG). Molecular dynamics (MD) simulations revealed the top ranked binding site for FA to be within the β-barrel, which is known to be a preferred binding site for hydrophobic ligands. The interaction is stabilized by three hydrogen bonds between FA and the protein side chains Lys69, Asn90, and Leu39, and hydrophobic π-alkyl interactions between the aromatic ring of FA and Val41, Leu58, and Ala86. However, the MD simulations revealed that FA was not fixed at this binding site, but that the interactions are more dynamic. Depending on the pH value, these interactions also seem to have a different influence on the protein’s conformation. The addition of FA at a low pH (2.4) results in the decrease of the α-helical structure by 3.9% and an increase of the β-sheet conformation by 5.1%, whereas the addition at a neutral pH (7.3) only results in a decrease of the α-helical structure by 0.5% [74]. In 2017, Jia et al. [75] also studied the binding of FA and β-LG using MD simulations. They detected seven amino acid residues taking part in the binding (Ala34, Gln35, Ser36, Trp61, Glu62, Asn63, and Gly64), of which four interactions were discovered to be hydrogen bonds (one with Gln35, two with Ser36, and one with Gly64). They also reported a conformational transition from α-helix to β-sheet resulting from the binding of FA [75]. These changes in the secondary structure are in accordance with studies described by Kang et al. [76], also showing a decrease of the α-helical structure of human serum albumin (HSA) with an increasing molar ratio of CA or FA to protein. However, they found a simultaneous increase of β-turn and random coil. Consequently, changes in secondary structure are dependent on the pH, as well as the ratio of PAs to protein [76]. Changes in secondary structure also entail changes in the protein’s solubility and temperature-dependent denaturation behavior [16,77]. Further, the non-covalent conjugation of proteins with PAs also affect hydrophobic/hydrophilic properties, and protein digestibility [27,28,29,30,37,38].

It becomes evident that not any single PAs always interact (non-covalently) with proteins in the same way, but that the molecular size and structure of both the PAs and the specific proteins dictate the interactions, and hydrophilic and hydrophobic interactions often occur simultaneously [68,69,71].

**Figure 7:**
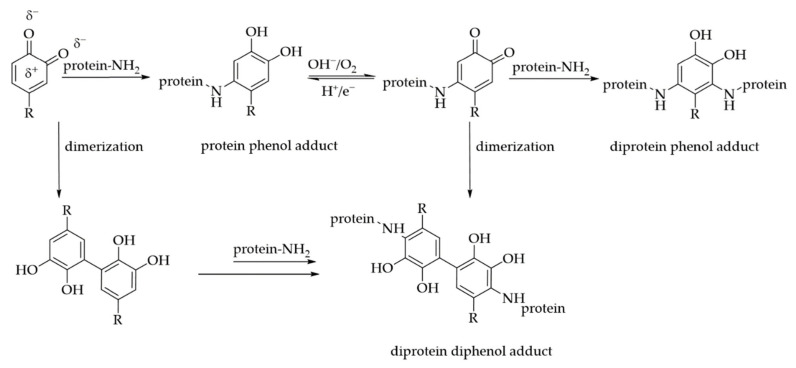
Exemplary crosslinking reaction between PAs (via quinones) and proteins (via lysine residues).

In comparison to non-covalent interactions, covalent bonding between proteins and PAs is often associated with a higher, basic pH, or is induced enzymatically via PPO or POD. These reactions take place via imine formation, Strecker degradation, or Michael-type addition, resulting in either Schiff bases, aldehydes, or Michael-type adducts [10]. These adducts have received special attention due to irreversible changes in protein properties and the possible formation of protein crosslinks. In a Michael-type addition, the reaction between the electrophilic *o*-quinones, deriving from the oxidation of PAs, and the nucleophilic side chains of selected amino acids results in the formation of different protein–phenol adducts [21,22,49,78]. Via the electrophilic reactive quinone intermediates, PAs are able to form covalent bonds with amino, sulfhydryl, thioether, phenolic, indole, and imidazole groups of the polypeptide side chains, involving the amino acids lysine, cysteine, tyrosine, methionine, tryptophan, or histidine as possible reaction partners [21,22,49,78,79]. The reactive quinones can either react directly with a nucleophilic protein side chain or undergo di-/oligomerization prior to the reaction with proteins. In both cases, the formed adducts can undergo reoxidation and subsequently react with another polypeptide [13,79,80]. Consequently, protein crosslinks are formed. Unlike non-covalent interactions, covalent bonds are almost irreversible. The possible reactions are illustrated in Figure 7.

In the context of covalent reaction modes, Prigent et al. [79] compared interactions of CGA with α- lactalbumin, lysozyme, and BSA at an alkaline pH (9) and acidic pH (5 and 6), catalyzed by PPO. Under both conditions, covalent bonds between the proteins and CGA were formed. However, at an acidic pH value, the peroxidase mediated interactions only occurred at pH 6, probably because of a limited PPO activity at pH 5. However, oxidation via periodate at pH 4 also resulted in the formation of covalent bonds. An addition of two molecules of CGA was mainly observed. For the lysozyme, an addition of up to four molecules could be detected, but the addition of a single molecule could not be observed. Thus, a dimerization in advance of the protein reaction is very likely [79]. Other studies also suggest the formation of phenolic acid dimers (or oligomers) prior to protein coupling, e.g., dimers of FA, CA, and *p*-coumaric acid, and that the dimers might be more reactive towards proteins than the monomers [80,81]. Then again, Rawel et al. [19] found evidence for the covalent addition of only one molecule of CGA bound to α-LA as well as β-LG at alkaline conditions.

Overall, three possible reaction products are conceivable: (1) the monomeric addition of a phenolic acid to a protein; (2) di- or oligomerization of PAs prior to the reaction with only one protein; (3) protein crosslinking via monomeric or oligomeric PAs.

There seems to be a difference regarding the quantity of PAs covalently bound to proteins, depending on whether the reaction in question is catalyzed by alkaline or enzymatic conditions.

In this context, Ali et al. [78] reported a difference in the proportion of bound rosmarinic acid (RoA) to alkaline/enzymatically-treated whey protein isolate (WPI). The sum of free amino and thiol groups of WPI modified with RoA was measured. While after alkaline treatment the sum of free groups was reduced by 45%, the enzymatic treatment only revealed a reduction of 36%.

The ability to crosslink proteins covalently depends on the type of PAs and the specific protein. Pham et al. [36] observed covalent crosslinking of flaxseed protein isolate by hydroxytyrosyl (HT), while FA only formed covalent bonds, but did not crosslink the proteins. As shown in Figure 4, FA bears a methoxy group at position 3 of the aromatic ring. This methoxy group enables only the formation of *o*-semiquinone, which is comparatively slower with regard to reactivity towards further compounds. However, other studies observed a certain crosslinking of a soy protein isolate or gelatin via FA [82,83]. Junwen et al. [84] used FA to crosslink caseins in an oxidative system consisting of hydrogen peroxide and horseradish peroxidase. They found improved emulsifying and gelation properties for the crosslinked caseins.

Similar to the non-covalent interactions, covalent bonding between PAs and proteins also results in conformational changes of the secondary structure [26,62]. For example, increasing amounts of BSA-bound PAs are accompanied by a loss of α-helical structures towards more disordered conformations [26]. The change in secondary structure may also cause changes in the protein’s functionality, e.g., the reduction of allergenic properties or, as in the case described above (casein + FA), altered emulsifying and gelation properties [35,84,85]. Regarding the allergenic potential, studies described by Lu et al. [35] indicate a reduced allergenicity for CGA-conjugated ovalbumin, a major allergen in egg white, resulting from conformational changes, which lead to an alteration of epitopes and therefore a decreased IgE binding capacity. A decreased allergenicity may be caused by conformational changes of epitopes and inhibition of the accessibility of linear binding sites by conjugation [86]. More directly, a conjugation of an amino acid in the sequence of an epitope can also affect allergenicity, mostly as complete inactivation of the epitope. The change in functional properties, such as allergenicity, derives from physicochemical alteration of the proteins upon covalent modification with PAs. This affects properties such as hydrophobicity/hydrophilicity, solubility and thermostability. Further, digestibility and enzyme activities can also be impaired. Consequently, other functional properties, such as gelation or foaming and emulsifying properties, are affected as well [25,26,31,32,33,34,36].

In addition to changes of protein properties, properties of the PAs can also be affected by the covalent and non-covalent interactions, e.g., antioxidant activity. Overall, both negative as well as positive effects of the antioxidant activity of PAs by association with proteins have been observed. Interactions with PAs enhanced the antioxidant capacity of the proteins [62,87,88,89]. Liu et al. [88] measured a higher antioxidant activity for covalent zein–CGA conjugates in comparison to non-covalent conjugates. However, the antioxidant capacity decreased in the phenolic–protein conjugates compared to free PAs, due to the limited number of reactive groups after conjugation [27,30,90]. In contrast, Fu et al. [91] found a higher antioxidant activity for CGA–gelatin conjugates compared to free CGA.

Transferring these interactions to food matrices reveals that the interactions between proteins and PAs are strongly influenced by chemical parameters (natural pH, enzymatic activity, type of protein, type of phenolic acid) as well as the processing conditions (temperature, adjusted pH, ionic strength). In most cases it is likely that a mix of interactions can occur [92].

## 4. Interactions of PAs with Carbohydrates

In addition to the interactions of PCs, especially PAs with proteins, interactions with starch or non-starch polysaccharides from plant cell walls are receiving increasing attention. The interactions of PCs with carbohydrates may influence technofunctional, physicochemical, and/or nutritional properties, such as digestibility, rheology, gelatinization, and retrogradation [40,93,94,95,96]. In general, PAs can react with various components by covalent or non-covalent bonds. The covalent bonds include interactions with non-starch polysaccharides, such as pectin, cellulose, and hemicellulose, via ester bonds [97]. PAs form non-covalent bonds with starch by forming complexes [41,95,98]. Naturally occurring ester bonds between starch and PAs have not yet been observed, but starch ferulates (starch esterified with FA) with different degrees of substitution have already been synthesized by esterification of starch with FA [99,100].

**Figure 8:**
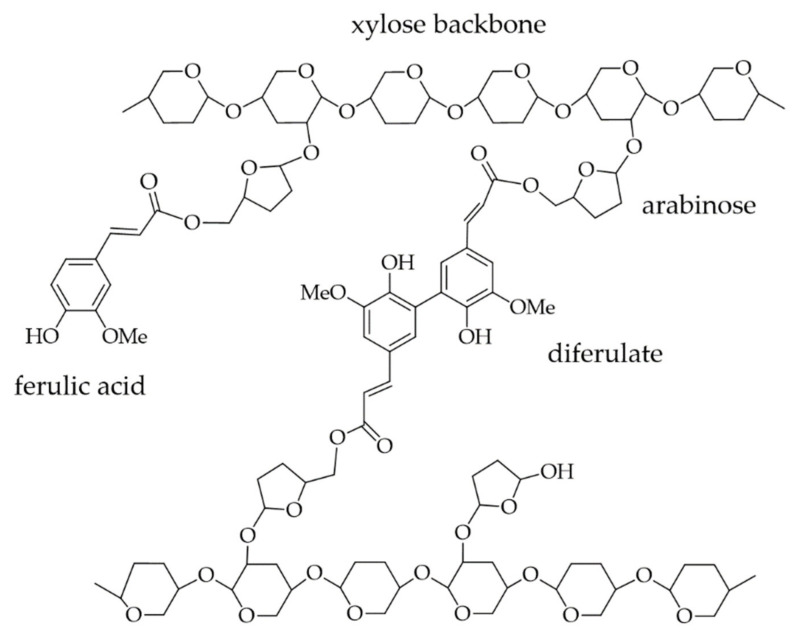
Structure of the ester bond of FA with AX.

The outer layer of cereals, such as maize, rye, and wheat, have been found to contain exceptionally high levels of bound and free FA [48,101]. As shown in Figure 8, FA is linked to arabinoxylan (AX), which is composed of a β-1,4-linked D-xylose backbone with substituted α-L-arabinofuranose units at C2- and/or C3-position, via an ester bond at the arabinose residue [102,103,104,105]. 

AX is classified into two different classes: water-extractable AX (WEAX) and water-unextractable AX (WUAX). While WEAXs are loosely bound to the cell wall, WUAXs are bound by either covalent ester linkages of FA and/or non-covalent bonds (hydrogen bonds, van der Waals forces) with AXs, proteins, and lignin [106,107]. Their solubility depends on the degree of substitution. A decreased arabinose-to-xylose ratio indicates a lower solubility [108]. In the cell walls of grasses, feruloylated AXs are the major non-starch polysaccharides [109]. As already mentioned in Section 2, ester-bound FA can also react by oxidative coupling, mediated by peroxidase, to form dehydrodiferulic acids, -triferulic acids, and -tetraferulic acids [57,58,110,111]. Thereby, primarily the 5-5′- and 8-O-4′diferulic acids are formed from the diferulic acids in their cyclic- and non-cyclic form [60]. In Figure 5 the different molecular structures of diferulic acids were shown already. These feruloylated wall-polymers can lead to oxidative crosslinking between other cell wall polysaccharides or proteins [58,112].

Zhu et al. [98] and Deng et al. [113] reviewed the non-covalent interactions between starch and PCs and their physical and nutritional effects. The non-covalent interactions between PCs and starch may be categorized into two complexes: (1) inclusion complexes (also called V-amylose complexes); and (2) non-inclusion starch complexes, in which intermolecular aggregates are formed by the interaction of the hydroxyl and carbonyl groups of the PCs with the starch constituents amylose and amylopectin via hydrogen bonds. The complex formations depend on the botanical origin of the starch, the chemical composition of the extract, the structure of the specific PCs, and the system’s pH, as altered by the possible addition of PAs [114,115]. Both complex types influence the technofunctional properties of starch and the polyphenols and can occur in all kinds of food processes [116].

Inclusion complexes are formed by hydrogen bonds, hydrophobic interactions, electrostatic, and ionic interactions. All are known to exist between PCs and the amorphous lamella of starch, with amylose forming a left-handed single helix structure, named V-amylose complexes [3,117]. The amylose helix has a hydrophilic outer surface due to the hydroxyl groups, while the inner surface of the helix is hydrophobic. Therefore, mainly hydrophobic interactions occur in this cavity. The structure and ligand complexation are stabilized by hydrogen bonds and van der Waals forces between the glucose residues, water molecules, and the ligands, like the PAs [118]. There have been several studies on the complexation mechanisms with amylose and complexation of amylose with a variety of small molecules, e.g., tea polyphenols, lipids, ibuprofen, or 2-naphthol, while the complexation of PCs is still rare [117,119,120,121,122,123,124,125,126,127,128]. Due to its longer and more linear chain length, amylose is more likely to form complexes with guest molecules compared to amylopectin. Consequently, starches with higher amylopectin contents often form fewer or no complexes with PCs. It must be mentioned that in some studies, the starch was debranched, or strictly linear amylose was used, so that the inclusion complex formation can occur [128,129]. Non-inclusion complexes were also identified, incorporating some of the guest molecules mentioned [129]. Due to the weak hydrophobicity, PAs are unlikely to form an inclusion complex with starch [130]. The amount and distribution within the group of Pas, as well as the limitation of the size of the cavity in the amylose helix, could also be a reason for diminished formation. It has been observed that larger guest molecules result in an expanded helix with more glucose residues [98,131]. The helix types vary from six to eight glucose units per helical turn. Larger phenolic substances preferentially form or require a helix of eight glucose residues [132,133]. The molecular size of the ligand determines whether a complex is formed. It is also crucial for identifying the amylose helix types [134]. Some studies illustrated that the binding of an aliphatic chain of, e.g., CGA, *p*-coumaric acid, or FA, formed an inclusion complex, but only the hydrophobic chain was incorporated [116,122,131,135]. Van Hung et al. [41] and Fang et al. [136] succeeded in their studies to produce a cassava debranched starch–FA complex. Their observations indicated that the FA was trapped in the hydrophobic core of the double helices formed between the linear amylose molecules. The advantage of these inclusion complexes is mainly that the included ligands are protected against other influences [137,138]. Li et al. [94,95] observed the formation of amylopectin complexes with CA, gallic acid, and FA, applying maize amylopectin and potato starch. However, it could not be identified whether those were inclusion or non-inclusion complexes.

**Figure 9:**
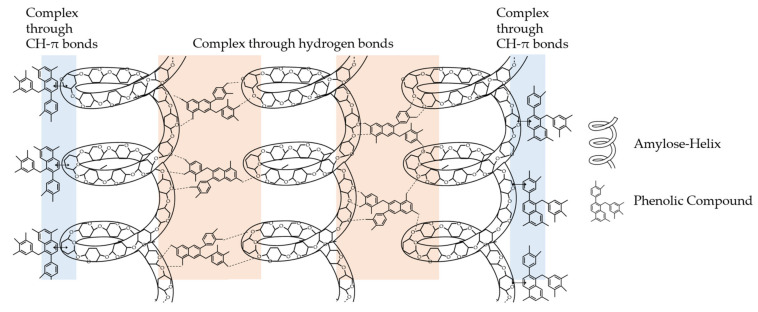
The model of non-inclusive complexes according to Guo et al. [129].

The interactions of the starch–phenolic non-inclusion complexes are also based on hydrophobic and electrostatic interactions, but the predominant binding forces are hydrogen bonds [3,139]. Guo et al. [129] reviewed non-inclusive complexes with green tea polyphenols. They provided a model of this non-inclusion complex in which numerous polyphenol molecules surrounded multiple starch helices via two to three weak CH-π bonds and hydrogen bonds (Figure 9).

There has been further investigation of these non-inclusion complexes for a better understanding and extension of this model. For example, gallic acid, FA, and CA were added to maize starch, as well as green tea polyphenols to lotus seed starch [93,96,129,140]. With the help of X-ray diffraction measurements, inclusion complexes could not be detected. Thus, the formation of a non-inclusion complex was concluded. However, a complete understanding of these interactions, the structure of the complexes, and the underlying nutritional effects has not yet been fully achieved.

## 5. Influence of Interactions between PAs and Proteins on Dough and Bread Properties

The natural occurrence of PAs in different cereals, such as wheat and rye, and their ability to interact with proteins, as mentioned above, raises the question of how their interactions affect the properties of wheat and rye products, especially bread.

As mentioned above, PA–protein interactions influence the protein’s physicochemical and biological properties. During the breadmaking process of wheat bread, the formation of dough and crumb is to a great extent dependent on the proteins; more precisely, on the gliadins and glutenins. During dough formation, these proteins build an elastic gluten network, being held together by intermolecular interactions. PAs may influence these interactions. Consequently, dough elasticity and viscosity, as well as bread quality, can be affected [141,142]. The formation and the properties of the wheat gluten network are crucial to the quality parameters of the resulting bread [143,144].

The stability of the gluten network relies to a great extent on the formation and reorganization of disulfide bonds between the glutenins and gliadins during the mixing and rising of the dough. However, PAs are likely to be involved in dough breakdown, by interfering with the disulfide bond formation, due to their reductive capacity. As a consequence, the amount of SDS extractable proteins increases [145,146,147]. Snelders et al. [148] observed an increased level of SDS-extractable high molecular weight polymeric gluten after the addition of FA. The addition of arabinoxylanoligosaccharides (AXOS) containing an equivalent amount of FA (2 to 18 g/kg flour) had the same effect. Thus, FA affects the gluten network, whether it is free or bound to AX. The reason for the increase of SDS-extractable proteins might be the reaction of FA with thiyl radicals derived from dough mixing. By interacting with the thiyl radicals the reformation of disulfide bridges is impeded and consequently the gluten network is weakened. However, the quantity of FA and different amino acids, such as cysteine or tyrosine, were not affected, which means that a covalent addition of FA to thiol groups from the cysteine residues, or hydroxyl groups from the tyrosine residues of the protein, did not take place during the baking process.

Although changes in dough rheology by the addition of FA or AXOS were observed (decreased resistance to extension and increased extensibility), final bread volume was not affected [148]. In contrast, other studies found that the addition of FA results in a diminished bread volume [147,149]. For example, Han et al. [147] observed that the addition of 250 mg FA/kg flour lowered the bread volume by about 4% and also decreased mixing time, mixing tolerance, and resistance to extension of the dough. Simultaneously an increase in SDS-soluble high-molecular-weight fraction was observed, probably due to reduction and reorganization of the gluten network, enhanced by the addition of PAs. In accordance with these results, Nicks et al. [149] reported a reduction of bread volume of 5–21% after the addition of 825–5000 mg FA/kg flour.

Disulfide bonds are usually found in three different conformations: *gauche-gauche-gauche* (*g-g-g*), *trans-gauche-gauche* (*t-g-g*), or *trans-gauche-trans* (*t-g-t*). Overmixing of dough causes a further rearrangement of disulfide bonds. The more stable *g-g-g* conformation decreases, while the amount of disulfide bonds with the energetically less stable *t-g-t* conformation increases. The addition of different PAs resulted in a shift in the balance of these conformations, even before dough breakdown, towards a higher amount of energetically less stable disulfide bonds, suggesting the incorporation of PAs in the three-dimensional protein network. This results in the formation of a less stable gluten network [150]. Additionally, PAs also had an influence on the amount of free sulfhydryl groups. Before dough breakdown, the addition of cinnamic and CA resulted in a diminished amount of free thiol groups compared to the control, accompanied by an increase in the less stable *t-g-g*- and *t-g-t* conformation for disulfide bonds, indicating the formation of aggregated structures during gluten formation [150]. This suggests the formation of covalent bonds. In contrast, the addition of FA showed an increase of free thiol groups during mixing before dough breakdown.

Although the effects of PAs on gluten networks have been intensively studied, the exact mechanism is still not comprehensively understood, and so it is difficult to predict the effect of the different PAs on dough and bread properties. Overall, PAs can reduce dough mixing time, dough strength, and accelerate dough breakdown, due to an influence on the transformation of disulfide bridges [145,146,147,148].

Different studies presented contradictory results on whether crosslinking between PAs and the newly formed thiol groups occurs. While Snelders et al. [148] did not find evidence for a crosslink between FA and thiol groups during dough breakdown, Huang et al. [151] reported a decrease in disulfide bridges, accompanied by a simultaneous decrease in free thiol groups after the addition of 10–40 g FA/kg of a model dough, indicating the occupation of free thiol groups by FA. As early as 1986, Jackson and Hoseney [152] found evidence for the formation of covalent cysteine–FA adducts in wheat doughs. Furthermore, Huang et al. [151] found a decrease of the denaturation temperature and enthalpy for model doughs following the addition of FA in comparison to the control. These results suggest an alteration in the gluten network’s spatial structure towards a more disordered weaker gluten network which tends to denature more quickly.

In addition to the disruption of the gluten network, resulting from the interaction of PAs with disulfide bridges and thiol groups, hydrogen bonding as well as π-alkyl and hydrophobic interactions between PAs and the gluten network can also influence its properties.

Krekora et al. [150] found evidence for the incorporation of PAs into the gluten network. FT-Raman spectroscopic studies revealed that the addition of cinnamic acid, coumaric acid, or FA initiated an increase in the intensity of the bands characteristic for tryptophan before dough breakdown. It had no influence on the intensity of the bands characteristic for tyrosine, suggesting an incorporation of the PAs into the gluten network by hydrophobic interactions with tryptophan. After the dough breakdown all PAs studied showed an increase in the bands characteristic for tryptophan.

In addition to the formation of a gluten network, the process of oxidative gelation is of importance for the dough and its baking properties [153]. During the process of oxidative gelation, water-soluble pentosans, mainly AX, form intermolecular networks capable of holding a high amount of water, as already mentioned (c.f. Section 4) [154,155]. These intermolecular networks are formed by the crosslinking of AX-bound FA via diferulates. The crosslinking is enabled by the presence of free radicals. An example of the resulting network is illustrated in Section 4. Next to the formation of AX networks, a reaction between AX-bound FA and proteins, likely via FA-tyrosine bridges, may also occur [156]. Piber and Koehler [157] found evidence for covalent crosslinking between dehydroferulic acid and tyrosine in rye and wheat breads. This crosslinking originates most likely from proteins of the prolamin fraction. In wheat, an increase of 300% of dehydroferulic acid-tyrosine was found in the course from the dough to the final bread. For rye, the increase was only around 25%. However, the amount of the dehydroferulic acid–tyrosine conjugates (DFT) in wheat dough only amounted to 0.5% of ferulates and 0.013% of tyrosine present in the flour. Consequently, the DFTs probably do not have a significant influence on dough functionality. Nevertheless, FA and its derivatives as well as other PAs might form similar conjugates. This assumption is supported by the fact that 24% of the added FA reacted with the wheat prolamin fraction.

The phenomenon of oxidative gelation is especially important for soft wheat or rye products, in which the formation of a gluten network is not primarily responsible for the structure formation of dough and bread.

During fermentation and baking, the content of PAs, especially the ubiquitous FA, increases in comparison to the wholemeal breads of wheat and rye. After baking, the release of bound FA into the bread is higher in the crumb in comparison to the crust [158,159]. Tian et al. [160] found that the fermentation and baking process increased the content of soluble PCs, as well as the antioxidant activity, in wheat bread. The process also slightly increased the content of insoluble PCs by releasing them from other matrix components. However, the amount of FA and diferulic acid isomers was not significantly influenced by the baking process.

Table 2 shows an overview of the influence of added FA on the behavior and functionality of wheat dough and bread from different studies.

In addition to the direct influence of PAs on dough and bread properties by incorporation into the gluten-network, PAs can also inhibit enzymatic digestion by blocking binding sites of proteases or interacting with endogenous enzymes [159,161]. The enrichment of wheat bread with PCs from green coffee beans diminished the digestibility of the proteins of the final bread. After in vitro digestion and electrophoretic characterization of the samples, phenol–protein interactions were confirmed of increasing intensities for protein bands depending on PC concentration [161]. A diminished digestibility of proteins can lead to a decreased nutritional value, as enzymes are unable to hydrolyze peptide bonds and thereby release indispensable amino acids. Furthermore, PAs, such as FA, are capable of inhibiting important enzymes, such as α-amylase and α-glucosidase [162], and consequently influence starch degradation.

Similar to wheat, enzyme activities in rye are also affected by the presence of PAs. During rye breadmaking, cinnamoyl, especially feruloyl esterases, control the release of insoluble and soluble ester bound PAs. The activity of these esterases is related to dough pH value. Increasing acidity inhibits enzymatic hydrolyzation. Xylanase activity negatively correlates with the amount of PAs. Consequently, arabinoxylan-hydrolyzing enzymes (endo-xylanase and xylosidase) can be inhibited by free PAs as well as by PAs bound to soluble and insoluble AX [159].

During breadmaking, the content of free FA increases in sourdough fermented rye breads, mainly due to the activity of esterases during the fermentation of rye doughs. However, it was found that after in vitro digestion of sourdough fermented rye bread with a mixture of simulated salivary, gastric, and pancreatic fluids, respectively, the amount of free FA increased further by about 22.5%, compared to non-hydrolyzed bread [163]. This increase after hydrolysis suggests the involvement of the PAs, especially FA, with other components, such as proteins or polysaccharides (e.g., AX or starch), through covalent or non-covalent interactions [41,42,151,152,162].

FA and diferulic acid derivatives, as shown in Figure 5 (mainly 8-*O*-diFA, 5-5-diFA, 8-5-diFA, and 8-5-benzofuran-diFA) are the most abundant PAs in rye, followed by sinapic acid and *p*-coumaric acid. Out of the total phenolic content, FA and the dehydrodimers make up about 68.5% and 24%, respectively [47].

In contrast to wheat, dough formation in rye does not primarily depend on the proteins, but rather on pentosans (mostly AX) and starch, as rye prolamins and glutelins do not form a continuous gluten network. It is assumed that the formation of such a network is hindered by the presence of rye pentosans. Furthermore, rye prolamins and glutelins have a different structural and quantitative composition in contrast to wheat gliadins and glutenins. Rye prolamins and glutelins (also called secalins and secalinins) are mostly homologous to wheat gliadins, with one exception: the 75k-γ-secalins. These proteins have a significantly higher molecular weight, and the N-terminal domain consists of additional repetitive sequences with a high content in glutamic acid, proline, phenylalanine, and tyrosine. The C-terminal sequence is homologous to γ-gliadins, with eight analogous cysteine residues. In contrast to the monomeric γ-gliadins, 75k-γ-secalins possess a cysteine residue in the N-terminal domain at position 12. This cysteine residue distinguishes the 75k-γ-secalins from γ-gliadins and probably is responsible for its dimerization via one intermolecular disulfide bridge. The limited number of free thiol groups might also hinder protein polymerization [164,165,166,167,168,169]. However, information on the influence of rye proteins on bread quality and sensory properties, as well as interactions with PCs, is scarce. Some studies suggest that protein quantity and thermodynamic behavior affect the sensory quality and volume of rye bread [170,171]. Overall, the interactions of PAs with wheat and rye proteins seem to have an impact on the stability of protein–protein interactions, protein denaturation, enzyme activities, and therefore quality parameters of the dough and the final bread. In both cases, covalent, as well as non-covalent, interactions must be considered, due to the prevailing chemical conditions. In wheat dough the pH ranges between 5.6–6.5, while the pH in fermented sourdough typically ranges between 3.5–5.4 [172,173,174,175]. Regarding the rather acidic pH, non-covalent interactions should prevail. However, due to the PPO and POD activity in wheat and rye, covalent interactions are also conceivable [176,177].

## 6. Influence of Interactions between PAs and Carbohydrates, Especially Starch and Pentosans, on Dough and Bread Properties

Compared to PC– and PA–protein-interactions, there are currently a comparatively lower number of studies on the influence of the interaction between PAs and starch or non-starch polysaccharides in dough or bread. However, the interaction between starch and PAs or other PCs has promising effects on nutritive properties, such as digestibility. PCs are able to inhibit digestive enzymes, such as α-amylase or α-glucosidase. Therefore, starch is not or is only partially degraded into minor sugar units, resulting in a low glycemic index after consumption of starch-rich foods and PAs. Consequently, PA-enriched bread or further cereal-based products could be used for special consumer groups, such as diabetics or obese people [115,178,179]. Besides this positive effect, the rheology, especially bread’s textural properties, are also affected by such interactions. When adding PCs or PC-rich ingredients, the quality of cereal-based products could be improved by diminishing hardness or adhesiveness [39,180]. Bread quality parameters are influenced during the whole production process (dough preparation, fermentation, baking). During gelatinization, interactions occur between PCs and starch, affecting the retardation of retrogradation. This effect was observed in PC-enriched bread, as well as in commercial bread, in which the PAs occurs naturally [181,182,183]. Most studies on the influence on starch have been investigated with synthetic starch, potato starch, or maize starch, with different amylopectin/amylose ratios [93,115]. The adhesive and textural properties change due to the interactions with PAs. Furthermore, the associated change in the pH value of wheat starch suspensions was investigated. As expected, the pH was lowered by the addition of PAs: for FA, the pH was 2.95, while for the control bread (without added FA), the pH was 6.79 [39]. In the study described by Zhu et al. [39], the dependence of pH with peak viscosity, hot paste viscosity and final viscosity was determined. The peak viscosity indicates the water holding capacity of the starch. The hot paste viscosity, as the name suggests, indicates the viscosity of the heated starch; the final viscosity describes the ability of the starch product to form a paste or gel after cooking and cooling. While no significant trend was observed regarding pH and peak viscosity, there was a positive correlation between hot paste viscosity and cold paste viscosity with pH in the starch suspension, resulting from the addition of PAs. The type of starch, the ratio of amylose and amylopectin, and the PCs itself, as well as its concentration, influence viscoelasticity [39,41,184]. The addition of PCs to low-amylose maize starch increased viscoelasticity, while the addition to intermediate-amylose and high-amylose starch decreased viscoelasticity [180]. So far, there has been no clear explanation for this observation. On the one hand, the viscosity could be influenced by the formation of hydrogen bonds and van der Waals forces, or by the formation of starch–PA complexes. By comparison, another study observed that an inclusion complex between FA and debranched cassava starch did not affect viscosity [41]. Moreover, other studies on resistant starch and gelatinization temperatures were conducted [181]. Resistant starch is a starch whose digestion is limited in the small intestine; instead, resistant starch enters the colon and is metabolized there. There are three types of natural resistant starch: (1) starch, which is difficult for enzymes to access due to the complex with intact cells; (2) starch or granular starch, which is found mainly in foods that are richer in amylose, and is characterized by a particular arrangement of starch molecules; and (3) resistant starch, which results from retrogradation [185]. The inclusion and non-inclusion complexes had no impact on resistant starch content or gelatinization [181]. Although gelatinization temperatures (initial, peak, and final) were significantly higher compared to native starch, similar temperatures were also observed for debranched cassava starch without FA incorporated [41]. Starch retrogradation affects hardness and adhesion, depending on temperature, water activity, type of starch, and PCs and its concentration, and thus pH value [39,180]. In this context, PAs and their interaction with starch are evident in diminishing the hardness and stickiness of wheat starch by altering the externalities of retrogradation [39]. A similar study showed that the addition of tea polyphenols to rice starch inhibited its retrogradation [182]. Zhu et al. [98] and Jakobek [186] reviewed many different starch-type interactions and described their influences on gelatinization, rheology, gelling, and retrogradation. There is still great potential for further studies in the field of PA addition, especially with regard to manufacturing processes and the associated storage, fermentation, and baking conditions.

The influence of PA–AX interactions is highly process-dependent. Before the grain can be used for bread production, the germ and the husk are removed and then milled. During this process, the PC content can be significantly diminished, as the PCs are located in the outer layers of the grains. Wholemeal wheat flour is the only type of flour that is milled entirely, partially avoiding a loss. In comparison, rye is regularly processed as wholemeal flour. During storage of the flour, the composition can change significantly and affect the interaction behavior. Conditions such as humidity, enzyme activity, pre-drying, and storage temperatures play an important role. Comparing fresh and six months-stored flours, the PA profile remained the same, but the stored samples contained only one third of the total phenolic content due to oxidation processes [187]. However, the oxidation processes and temperature increase could also affect the oligomerization of ferulates or release of some ester-bound FA, which increases the antioxidant capacity [61,188]. Cheng et al. [189] reported that milling and the resulting lowering of the particle size increased the amount of PAs that could be released from the bran. However, the storage of milled fractions for an extended time resulted in a loss of antioxidant activity in wheat.

Wheat and rye flours can be used for various products, especially bakery goods. During all processes, many interactions of PAs, especially with pentosanes, can take place. To better understand these interactions, it is important to explain the formation of dough in wheat and rye. Baking properties are ensured in wheat by the gluten proteins and in rye by the swellable pentosans and glycoproteins. Rye flour is mainly processed as a sourdough process, employing lactic acid bacteria or by adding organic acids. A more acidic environment (4.0–5.5) results in a higher water-binding capacity of the proteins and pentosans [190]. Furthermore, the water-binding capacity also depends on FA: the pentosans undergo crosslinking via FA and thereby contribute to gel formation, thus improving the mechanical properties of the dough. During gelling, a natural oxidative coupling of WEAX occurs, leading to a significant increase in viscosity and the development of a three-dimensional gel network. The oxidation process is ensured by the peroxidase naturally occurring in rye or by adding laccase, which allows the formation of di- or oligoferulates. However, hydrogen bonds might also be formed between neighboring AX chains that are close to each other due to the diferulate bonds [191]. The gel network is formed by intermolecular crosslinking through feruloyl groups of the AX molecules [192,193]. These advantages are often used in wheat dough when adding pentosans. More precisely, the WEAX increase the aqueous phase’s viscosity and provide stability of the retained gas in the network by forming a liquid film around the gas cells of the fermented dough, and the protein foam is ensured [194]. Consequently, the protein foam is thermally more resistant and the gas is retained; thus, the bread loaf keeps its volume [192].

However, WUAX destabilize the gas by causing breakdowns in the cavities during fermentation. By adding endoxylanases and arabinofurosidases, WUAX can be solubilized to improve viscosity, decrease stiffness, as well as increase hydrogen bonding [195]. These enzymes can destroy the backbone of the AX, thereby exposing regions that contain esterified FA. Nevertheless, pentosans added to wheat dough form a secondary network in addition to the gluten network, which possibly occurs by the formation of crosslinks with other WEAXs or the gluten proteins via diferulates [195]. However, WUAXs do not only have adverse impacts: they tend to swell strongly, and the crumb remains moist during the baking process. As already implied, the type of fermentation, the use of microorganisms, and the addition of enzymes are very crucial, as they affect the interactions between PAs/PCs with carbohydrates, which have an impact on the bioavailability, antioxidant capacity, or product quality of the baked goods, such as loaf volume and typical crumb structure [196]. In a study described by Boskov Hansen et al. [47], sourdough fermented rye bread was imitated by adding acetic and lactic acid instead of lactic acid bacteria. Free FA content of 3 µg/g d.m. was initially determined in the whole rye flour. After dough preparation and fermentation, a content of 12 µg/g d.m. was determined. It is possible that fermentation leads to hydrolysis of the ester bonds between AX and FA, and therefore an increase in free FA can be detected. The endogenous enzymes were almost inactivated, probably due to the acidic environment. Moreover, no change in diferulate concentration was detected, although it would be very likely that this could result from peroxidase-catalyzed oxidative coupling. Due to the oxidative crosslinking, a significant increase in viscosity is observed [155,197]. Konopka et al. [158] investigated the influence of breadmaking processes in wheat and rye with regard to FA availability and antioxidant properties. The fermentation with baker’s yeast and sourdough was compared and the changes in FA content in flour, dough, and bread were studied. In direct comparison, the rye flours, doughs, and bread obtained higher PCs and free FA contents than wheat flour, dough, and bread. Nevertheless, free PAs increased during the fermentation and baking in wheat as well as rye bread. In similar studies with sprouted whole grain flours, even more free PAs after fermentation and baking were detected [173]. The pH reduction due to sourdough fermentation leads to an optimal environment for hydrolases. Consequently, these cleave the esters and glycoside bonds of the PA–AX, and thus release the single compounds [47,158,198]. Skrajda-Brdak et al. [163] subsequently extended the study described by Konopka et al. [158] using different yeasts. However, they were unable to gain further significant knowledge about fermentation. In addition to fermentation, several parameters, such as water and oxygen content and temperature, impact the fermentation kinetics and the solubility of PAs [158,199]. An optimum pH value of 7 and a suitable starter culture may enhance the activity of rye cinnamoyl esterase. This enzyme, usually in combination with arabinofurosidases, cleaves PAs crosslinks, which could explain the increase in FA content [47,173]. An extension of the dough fermentation time led to a significant increase in antioxidant capacity [200]. Sourdough fermentation resulted in a significant increase in this effect in wheat and rye dough, in the flour, crust, and crumb of both types of rye bread (yeast-fermented and sourdough-fermented), and the crust of white wheat bread [158,163].

Heat or extending baking time changed the spectrum of antioxidant substances. Destruction of previously antioxidant compounds, possibly FA–AX interactions, occurred as well as the formation of new substances, e.g., Maillard products [200,201]. Regarding antioxidant capacity, increased antioxidant activities could already be determined after baking, due to an increased temperature [158]. With increasing temperatures, it is often observed that the content of bound PCs decreased, while the content of free PCs increased [188,202,203]. Further increase of different heating time and temperature enhanced this effect [188,204]. However, it is obvious that these two parameters seemed to have a decisive influence on the release of PAs. In a study described by Menga et al. [205], the increase in bound vanillic acid, p-coumaric acid, salicylic acid, and FA was intensified, when baking at 220 °C for 30 min. Thus, the thermal process can be assumed to support the release as well as the oxidation reactions forming quinones, with subsequent redox reactions [158,205]. Consequently, the covalent bonds to AX are formed. However, when the dough is subjected to too much heat stress, the cell wall of the cereals can break down, enzymes are released and denatured by the heat. Then, the oxidation process does not continue [188]. An increase in free PAs is not always accompanied by a decrease in bound FA. Due to the thermal process, it is also possible that larger polyphenols, such as tannins, decompose to simple PAs [189].

To understand the influence of the interactions between starch or pentosans with PAs, further studies on the complexes must be carried out. The type of starch or PCs and the manufacturing parameters affect the interactions, which are not yet fully understood. In contrast, the influence of AX–PA interactions is better elucidated. However, further studies on the influence of the respective baking processes could be conducted as well.

## 7. (Ternary) Interactions between PAs, Proteins, and Carbohydrates, and Their Influence on Dough and Bread Properties

Regarding food chemistry and the technology of bakery products, proteins and polysaccharides and their interactions with PAs determine processing and product properties. It has been demonstrated that the interactions of PAs with proteins or carbohydrates are based on similar mechanisms. Consequently, it is possible that interactions between all three components can occur, and that these ternary interactions are even more likely to influence dough and bread properties.

**Figure 10:**
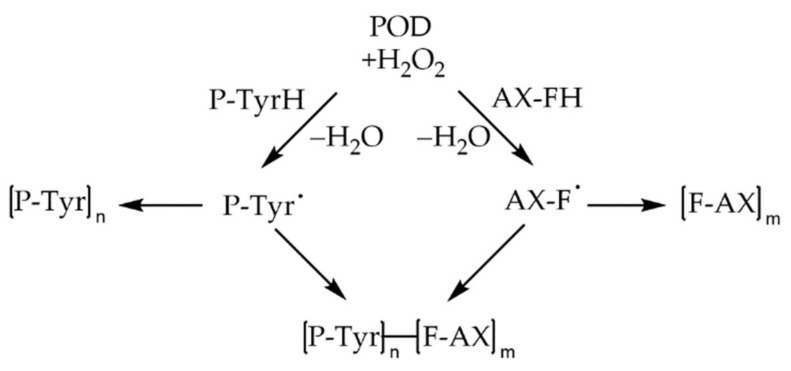
Proposed mechanism of the chemoenzymatic crosslinking between proteins and AX via reactive tyrosine and FA residues. Modified from Boeriu et al. [207].

As already mentioned, rye AX plays a major role in the technofunctionality of dough and bread. In comparison to wheat, the content of AX in rye is almost twice as high [206]. Buksa et al. [44] found evidence for AX–protein complexes crosslinked via FA in a rye model bread. Isolated AX–protein complexes were associated with the highest bread volume. They concluded that the complex formation might be enzymatically mediated by peroxidase. Additionally, they also considered the involvement of hydrogen bonds [44]. These types of complexes were also observed in a model consisting of feruloylated wheat-AX and β-casein, catalyzed by horseradish peroxidase. Size exclusion chromatography with UV-detection at 280 nm (characteristics for polymers containing aromatic residues) and 320 nm (characteristic for C–C aromatic linkages) confirmed a complex formation with absorbance at both wavelengths. The reaction was proposed to occur via reactive FA and tyrosine residues [207]. Indications of the presence of such crosslinks in wheat and rye were already given in 2005 by Piber and Koehler [157]. The proposed mechanism of the formation of protein–FA–AX complexes is shown in Figure 10.

Revanappa et al. [208] studied the effect of peroxidase on textural qualities of wheat dough. They found that the addition of peroxidase decreased dough adhesiveness. They concluded that an increase in molecular weight of AX is responsible for improved dough properties and found an increase in protein and FA content in AX fractions. Consequently, peroxidase-mediated crosslinking via FA between AX and proteins might be responsible for the increase in molecular weight.

Elofsson et al. [209] found evidence for electrostatic interactions between extracted secalins and AX fractions of rye. However, the presence of FA or other PAs in the AX fraction was not discussed.

The formation of ternary covalently linked conjugates between FA, proteins, and carbohydrates was also observed in other biomaterials [210,211]. For example, 8-5′ non cyclic diferulic acid was identified as being covalently linked to carbohydrates of the arabinogalactan–protein fraction of gum arabic [210]. Wang et al. [211] studied the effect of laccase-induced covalent complexation of β-LG, FA, and chitosan on β-LG properties. Free amino groups in β-LG decreased during complex formation with a rising laccase concentration. Additionally, the interactions revealed a loss in α-helical and β-sheet conformation and an increase in unordered structure, exposing amino acids, such as tyrosine, which also has the ability to form covalent crosslinks with FA under the influence of oxidases, as shown in Figure 10. The denaturation temperature and enthalpy of β-LG increased after complexation from 90.50 °C to 97.01 °C and 51.95 J/g to 66.52 J/g, respectively.

There is an interest in the food industry in heterocrosslinking of proteins with polysaccharides, as these biopolymer-conjugates reportedly enhance technofunctional properties, such as the stabilization of emulsions or foams [212,213].

For covalent crosslinking, the availability and type of PPO should not be underestimated, as they have different capacities to oxidize reactive sites, e.g., laccase showed better results in the formation of hetero-conjugates between oat spelt xylan, PAs, and α-casein compared to tyrosinase. However, both oxidases were able to form the corresponding reaction products [55].

Covalent crosslinking of water soluble feruloylated AX (WEAX) during oxidative gelation, as shown in Figure 10, is associated with enhanced elasticity, gelling and a high water retention ability. As already mentioned, WEAXs are also able to interact with proteins via PAs. To study the effect of protein on the gelation capacity of WEAX gels, Vansteenkiste et al. [191] utilized BSA as a model protein. By addition of BSA, the elastic module (*E*’) of the WEAX gel decreased. However, an increase in FA dimerization was not observed. Therefore, the interactions between WEAX and BSA are most likely of a non-covalent nature. On the other hand, the interactions hindered the enzymatic degradation of BSA, even after heat treatment.

As polysaccharides often exceed the size of proteins, their solubility also contributes to a large extent to those of the resulting complexes. Depending on whether the polysaccharides are water-soluble or insoluble, the properties of the complexes can also differ as can their effects on food functionality. As mentioned above, protein–PA conjugates with WEAX were found in wheat and rye. Consequently, the formation of complexes with WUAX would also be conceivable. In general, WEAXs are associated with good dough and bread qualities, whereas a high amount of WUAX is considered to be disadvantageous [195,214]. As the presence of AX–protein complexes crosslinked via FA in model rye breads was correlated with the highest bread volume, it is possible that such complexes with WUAX may negatively influence dough and bread properties. Recently, it was possible to show that water insoluble non-starch polysaccharides (NSPS) and proteins in rye and their interactions play a role with regard to bread quality, as protein denaturation and starch gelatinization are partly inhibited, accompanied by a higher amount of NSPS for wholemeal samples, resulting in poor bread quality [171]. A limited starch gelatinization might occur due to the starch’s competition for water with other components, such as pentosans or proteins, similar to wheat, where gluten proteins and starch compete for water, which can influence dough and bread strength [215,216,217]. Furthermore, the formation of protein layers surrounding the starch granules can hinder water transport and hydration of starch granules, and consequently lead to inadequate gelatinization [218,219]. Besides the limited water transport, protein–FA–polysaccharide complexes also inhibit protein denaturation, and hence limit the amount of released free water necessary for the gelatinization of starch during the baking process. This could lead to the phenomenon of dry-baking, which is well known for rye breads. However, the strength/ratio of contribution of PAs in these biopolymer interactions is not clear. Nevertheless, as they are naturally associated with AX and can interact with proteins and polysaccharides via hydrophobic, hydrophilic, and covalent forces, their involvement seems to be obvious.

In conclusion, the interactions between PAs, especially FA with proteins and polysaccharides, have an impact on dough elasticity, viscosity, and final bread quality. In wheat, it is primarily the gluten network consisting of proteins that is affected by interactions with PAs, some of which may also be bound to pentosans. In addition, in wheat and rye, interactions of PAs with endogenous enzymes affect starch degradation and hydrolyzation of pentosans. In contrast to wheat, it appears that in rye simultaneous interactions between PAs with protein, as well as polysaccharides and combinations of all three, play a major role. An example would be the formation of AX–FA–protein complexes, as proposed in the model shown in Figure 10. The formation of ternary complexes can impact dough performance and baking quality. The complex formation of rye proteins with feruloylated WEAX seems to have a positive influence on the bread volume. However, interactions with insoluble components, such as WUAX, might have the opposite effect. Such complexes may influence water availability and starch gelatinization and therefore have a negative impact on bread quality (dry-baking). Nevertheless, the implications and insights into chemical mechanisms and conditions need to be explored further in order to make accurate predictions regarding these interactions.

## Figures and Tables

**Table 1:** Overview of the binding capacities for FA and BSA and the corresponding methods.

K-Value [mol^−1^ dm^3^]	pH (Temp)	Method	References
1.793 × 10^4^ 25.47 × 10^4^ 80.68 × 10^4^	7.2 (25 °C) 7.2 (35 °C) 7.2 (45 °C)	Fluorescence quenching (tryptophan)	[69]
17.24 × 10^3^ 4.01 × 10^3^	4.8 (25 °C) 7.0 (25 °C)	Hummel–Dreyer method (HD)	[23]
5 × 10^3^ 5.56 × 10^4^	4.8 (T n.a.) 7.0 (T n.a.)	Fluorescence quenching (tryptophan)	[68]
40.15 × 10^4^	7.4 (25 °C)	Fluorescence quenching (tryptophan)	[37]
5.56 × 10^4^ 5.09 × 10^4^ 2.49 × 10^4^	7.8 (20 °C) 7.8 (25 °C) 7.8 (25 °C)	Affinity capillary electrophoresis (ACE) Fluorescence quenching (tryptophan)	[71]
5.1 × 10^4^	8.5 (T n.a.)	Surface plasmon resonance (SPR)	[70]

T n.a. = temperature not available.

**Table 2:** Influence of added FA on the behavior and functionality of wheat dough and bread.

Effect	FA Added	References
Increased dough strength Reduction of mixing time	0.034 g/100 g	[145]
Increased amount of SDS-soluble proteins in dough Reduction of bread volume Reduction of mixing time Decreased dough strength Increased crumb hardness	0.025 g/100 g	[147]
Increased number of SDS-soluble proteins in dough Reduction of bread volume Increased crumb hardness	0.0825–0.5 g/100 g	[149]
Decreased amount of disulfide bonds No influence on bread volume	0.2–1.8 g/100 g FA/eq. AXOS-FA	[148]
Decreased amount of disulfide bonds Decreased amount of free thiol groups Decreased denaturation enthalpy and temperature of gluten proteins	1–4 g/100 g	[151]
Decreased amount of disulfide bonds Increased amount of free thiol groups	0.05–0.2 g/100 g	[150]

## Data Availability

Not applicable.
